# Facial Fabrication and Characterization of Novel Ag/AgCl Chloride Ion Sensor Based on Gel-Type Electrolyte

**DOI:** 10.3389/fchem.2020.574986

**Published:** 2020-11-03

**Authors:** Seil Kim, Gwangryeol Park, Hong-Ju Ahn, Bung Uk Yoo, In-Hyuck Song, Kyu-Hwan Lee, Kwang Ho Kim, Jae-Hong Lim, Joo-Yul Lee

**Affiliations:** ^1^Department of Electrochemistry, Korea Institute of Materials Science, Changwon, South Korea; ^2^School of Materials Science and Engineering, Pusan National University, Busan, South Korea; ^3^Department of Engineering Ceramic, Korea Institute of Materials Science, Changwon, South Korea; ^4^Advanced Materials Engineering, Korea University of Science and Technology, Daejeon, South Korea; ^5^Department of Materials Science and Engineering, Gachon University, Seongnam, South Korea

**Keywords:** chloride ion sensor, Ag/AgCl electrode, diatomite ceramic membrane, gel-type internal electrolyte, reinforced concrete structure, chronopotentiometry

## Abstract

In this study, a novel chloride ion (Cl^−^) sensor based on Ag wire coated with an AgCl layer was fabricated using a gel-type internal electrolyte and a diatomite ceramic membrane, which played an important role in preventing electrolyte leakage from the ion-selective electrode. The sensing performance, including reversibility, response, recovery time, low detection limit, and the long-term stability, was systemically investigated in electrolytes with different Cl^−^ contents. The as-fabricated Cl^−^ sensor could detect Cl^−^ from 1 to 500 mM KCl solution with good linearity. The best response and recovery time obtained for the optimized sensor were 0.5 and 0.1 s, respectively, for 10 mM KCl solution. An exposure period of over 60 days was used to evaluate the stability of the Cl^−^ sensor in KCl solution. A relative error of 2% was observed between the initial and final response potentials. Further, a wireless sensing system based on Arduino was also investigated to measure the response potential of Cl^−^ in an electrolyte. The sensor exhibited high reliability with a low standard error of measurement. This type of sensor is crucial for fabricating wireless Cl^−^ sensors for applications in reinforced concrete structures along with favorable performances.

## Introduction

Reinforced concrete (RC) plays a critical role in the construction industry, especially in the construction of buildings, bridges, and port facilities, because it has higher tensile and compressive strengths than other building materials (Javadian et al., 2020). Steel is the most commonly used reinforcement material for RC structures because it has high tensile strength. However, steel reinforcements undergo corrosion because of chloride ions (Cl^−^), which is a main cause of the failure of RC structures (Imounga et al., 2020). These ions promote the breakdown of the protective passive layer by decreasing the pH of the pore water, leading to the formation of corrosion pits in metals (Li et al., 2017). Therefore, Cl^−^ monitoring in RC structures is a key strategy to prevent the corrosion of reinforced steel. The current techniques for determining the local Cl^−^ content in RC structures require destructive sampling methods such as drilling, grinding, and cutting. These methods are time-consuming and costly, and it is difficult to continuously obtain information at the same location over time (RILEM TC 178-TMC, 2002; Femenias et al., 2016). Furthermore, it results in additional indirect costs due to road closures and traffic delays (Torres-Luque et al., 2014). Therefore, several research groups are investigating the development of nondestructive *in situ* monitoring techniques to measure Cl^−^ ingress in RC structures, which can afford fast measurements with high reliability and real-time ingress profiles without destroying the structures (Abbas et al., 2013). Some existing nondestructive *in situ* monitoring methods include electrical resistivity measurement (Corva et al., 2020), fiber optic sensing (Bassil et al., 2020), potentiometry (Gandía-Romero et al., 2016), and chronopotentiometry (Abbas et al., 2013; Abbas, 2015). Among these, the chronopotentiometry technique based on an electrochemical method is employed for the indicative determination of Cl^−^ content in infrastructures, the principle of which is described as follows. In a three-electrode system comprising a counter electrode (CE), reference electrode (RE), and working electrode (WE), a constant current is applied to the WE and the potential response is measured with respect to the RE (Abbas, 2015; Dobbelaere et al., 2017; Raccichini et al., 2019). The as-fabricated Ag/AgCl ion-selective electrode (Ag/AgCl ISE) used in this method acts as the WE, which when embedded in the sensor device can be used to determine free Cl^−^ concentration near the rebar without changing the surrounding environment (Angst and Polder, 2014). This is because the electrode shows a Nernstian response to the variation in Cl^−^ or Ag^+^ activity and displays excellent sensitivity (Karthick et al., 2017). Here, the concrete has a strong alkalinity (pH 12.5–13.5). Therefore, the materials that are used as the electrolytes for RE should have alkali resistance, long-term chemical stability, and leak resistance characteristics. Conventional Ag/AgCl REs comprising a chamber and membrane based on glass are not suitable for the *in situ* measurements at the steel/concreate interface owing to their high alkalinities at the interface as well as fragility (Jin et al., 2014). Furthermore, traditional Ag/AgCl REs employ liquid-type solution as an internal electrolyte, which shows the drawback of continuous exudation of KCl internal solution. KCl exudation is one of the most important parameters for the reliable performance of traditional Ag/AgCl REs (Femenias et al., 2016). Solid-type electrolytes can decrease the rates of electrolyte loss and contamination and prolong the life span of REs. However, these suffer from low ionic conductivities, and the moisture content of the electrolyte varies with the change in concrete moisture, which causes the potential fluctuation of the RE, thus affecting the accuracy of corrosion monitoring in RC structures (Tang et al., 2015). To address this issue, a gel-type internal electrolyte and diatomite ceramic membrane (DCM) were employed in this study to fabricate the Ag/AgCl ISE, which allowed the application of the electrode in highly alkaline environments, such as RC structures, without the exudation of the internal electrolyte. In particular, the DCM could effectively prevent the leakage of the inner solution, which involved the dissolution of the AgCl coating layer. The aim of the present study was to fabricate a novel Ag/AgCl-based ISE comprising an Ag wire coated with AgCl with a gel-type internal electrolyte and DCM. Cl^−^ sensing characteristics included the response potential, reliability testing, response time, and recovery time of the sensor device, which were systemically determined in the electrolytes with different Cl^−^ contents. Finally, wireless sensing based on Arduino Uno was performed using the as-fabricated Cl^−^ sensor in KCl solution. This type of sensor is a key component for fabricating gel-type internal electrolyte-based Cl^−^ sensors with favorable sensing performances.

## Experimental

### Synthesis of Diatomite Ceramic Membrane

Diatomite (Celite 499, Celite Korea Co. Ltd., South Korea) and kaolin (Sigma-Aldrich, USA), as a strength enhancer material, were used to fabricate diatomite–kaolin composite membrane for sensor applications. The average particle sizes of the as-received diatomite and kaolin were 12.7 and 1.5 μm, respectively. To increase the sinterability, the size of the diatomite particles was decreased to a powder-to-volume ratio of 2:1 by ball-milling for 24 h using alumina balls. Diatomite and kaolin (weight ratio of 9:1) were subjected to ball-milling for 3 h to afford a power-to-volume ratio of 0.5:1. Finally, the diatomite–kaolin composite was dry-pressed at 18.7 MPa with polyethylene glycol as a binder, followed by sintering at 1,200°C for 1 h. The as-fabricated diatomite–kaolin composites were cut into cylinder-shaped samples of 1-mm diameter and 1-mm height.

### Synthesis of AgCl-Coated Ag Wire

An Ag wire with a diameter of 1 mm and purity of 99.99% was used to synthesize the AgCl-coated Ag wire. The experimental procedure for synthesizing the Ag/AgCl wire in this study is described as follows. First, prior to galvanostatic polarization (GP), the Ag wire was immersed in acetone (ACS reagent, >99.5%, Sigma-Aldrich, USA) with sonication for 10 min to remove the surface oil, followed by rinsing with distilled water. Next, the Ag wire was ground in one direction using a silicon-carbide abrasive paper (#2000 grit) to remove the native oxide layer and then dipped in acetone and 5% nitric acid (HNO_3_; 60%, Sigma-Aldrich, USA) solution for 10 min to remove the impurities. Thereafter, the Ag wire was cleaned in ethanol ultrasonically and dried in air. For GP, the WE terminal of the potentiostat was connected to the Pt-mesh and the CE terminal was connected to a pretreated Ag wire. After GP, an immersion process was employed to synthesize the AgCl layer on the surface of an Ag segment. The entire process was performed in 1 M hydrochloric acid (HCl; ACS reagent, 37%, Sigma-Aldrich, USA) at room temperature.

### Gel-Type Internal Electrolyte

The synthesis of the gel-type internal electrolyte is described as follows. First, 3.5 mol/L potassium chloride (KCl; 99.0%, Sigma-Aldrich, USA) solution was prepared and heated at 60°C. Then, 8 ml of glycerol (>99.5%, Sigma-Aldrich, USA) was poured into the KCl solution, which played an important role in preventing the high-density gelation of internal electrolyte. Finally, 5 wt.% of hydroxyethyl cellulose (HEC, 200–400 mPa·s, DaeJung Co., Ltd., South Korea) was slowly added into the heated KCl solution (100 ml KCl solution with 5 g HEC) with constant stirring at 150 rpm, affording the HEC-gelled electrolyte. The conductivity of the gel-type electrolyte was 226 mS/cm at 5 wt.% HEC and that of 3.5 M KCl solution was 294.8 mS/cm (Kim et al., 2017). This indicated that the gel-type electrolyte could decrease the migrating rate of ions, which further decreased the rates of electrolyte loss and exterior ions entering into the internal electrolyte. Furthermore, HEC showed good water absorptivity. Using the HEC-based gel-type electrolyte decreased the rate of moisture loss, thus prolonging the life time of RE in the RC structure.

### Fabrication of Ag/AgCl Ion-Selective Electrode

In this study, polyvinyl chloride (PVC) was used as the housing material because PVC is abrasion resistant and chemically resistant to many acids, bases, and corrosives, with good mechanical strength and toughness. First, a hole with a dimeter of 1 mm was melted at one end of each PVC chamber using a hot iron. Cylinder-shaped DCMs were then inserted into the holes. Next, the as-synthesized Ag/AgCl wire was inserted into each PVC chamber, leaving a space between the wire and membrane. Thereafter, 8 ml of the gel-type internal electrolyte was slowly poured into each PVC chamber, in which the Ag/AgCl wires were inserted. Finally, the top of each PVC chamber was sealed using a rubber cap and insulating tape. The as-fabricated Ag/AgCl ISE was stored in 1 M KCl solution.

### Characterization

**Figure 1:**
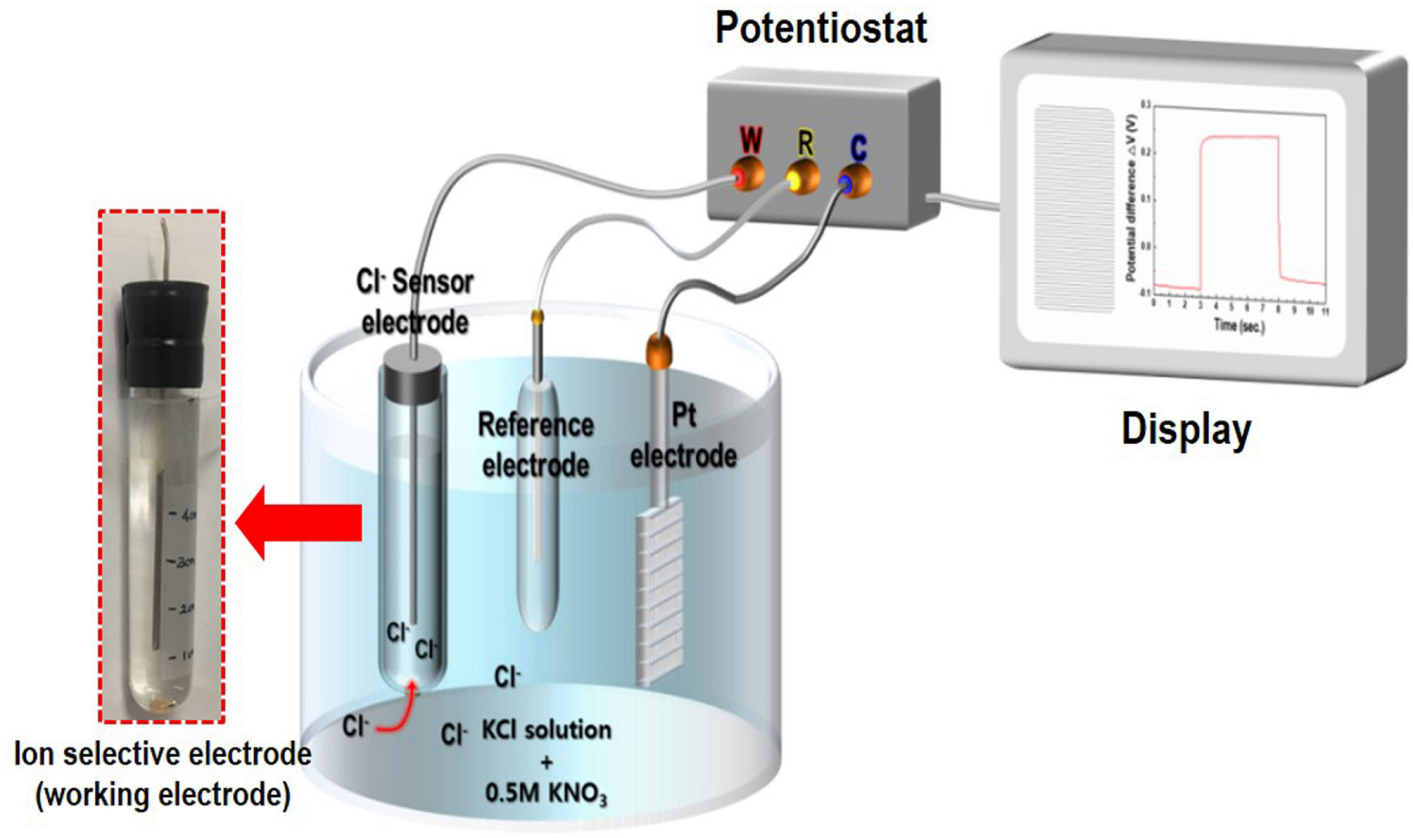
Schematic of the chloride ion sensor comprising an Ag/AgCl reference electrode, Pt-counter electrode, and Ag/AgCl ion-selective electrode (Ag/AgCl ISE) based on a gel-type internal electrolyte and diatomite ceramic membrane.

The crystal structure and crystallinity of the as-synthesized Ag/AgCl wire obtained by GP and immersion were determined using X-ray diffraction (XRD; Ultima IV CuK, Rigaku Corporation, Tokyo, Japan). X-ray photoelectron spectroscopy (XPS; Kratos Analytical Ltd., Manchester, UK) was employed to measure the changes in the surface composition of the Ag/AgCl wire. The morphology and composition were analyzed using field-emission scanning electron microscopy (FE-SEM; Hitachi S-4800, Hitachi Ltd., Tokyo, Japan) coupled with energy-dispersive X-ray spectroscopy (EDS; Oxford INKA X-Act, Oxford Instruments, Abingdon, UK). Cl^−^ measurement with the as-fabricated Ag/AgCl electrode was performed using a potentiostat (BioLogic, EC-Lab instrument, Seyssinet-Pariset, France) in electrolytes containing 0.5 M KNO_3_ and 10–600 mM KCl. The WE terminal of the potentiostat was connected to the as-fabricated Ag/AgCl ISE. The CE terminal was connected to the Pt-mesh, and the RE terminal was connected to the commercial Ag/AgCl RE. When a current pulse was applied between the WE and CE, Cl^−^ species near the WE were consumed, locally depleting the Cl^−^ near the surface of the WE, leading to a concentration gradient that resulted in a potential difference at the WE with respect to the RE (Bard and Faulkner, 2001). A schematic of the sensing measurement setup is shown in Figure 1.

## Results and Discussion

**Figure 2:**
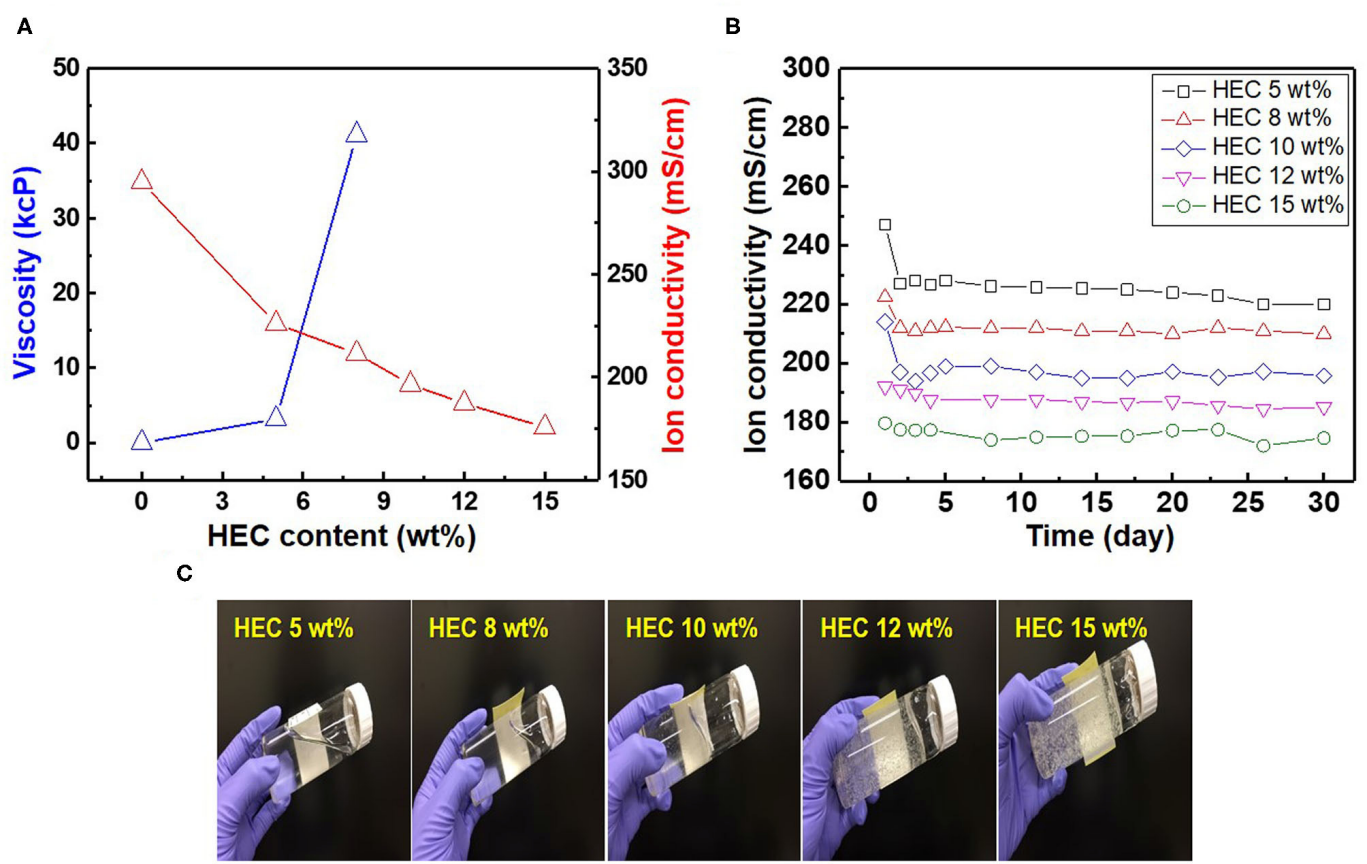
Relationship between **(A)** hydroxyethyl cellulose (HEC; wt.%), viscosity, and ion conductivity, **(B)** time-dependent ion conductivity, and **(C)** optical images of gel-type electrolyte with different HEC contents.

**Figure 3:**
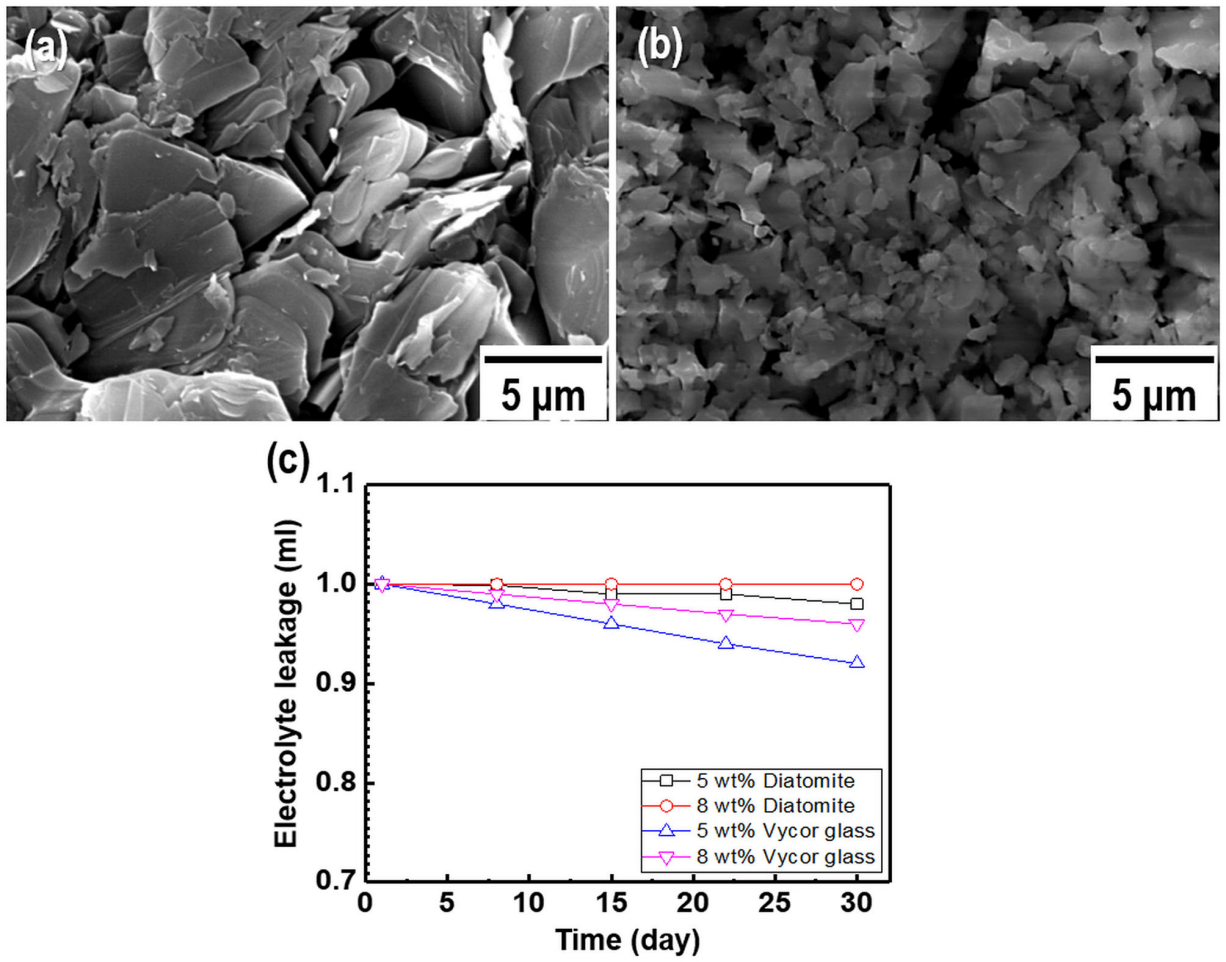
Field-emission scanning electron microscopy (FE-SEM) images of two types of membranes: **(a)** Vycor glass membrane and **(b)** as-synthesized diatomite ceramic membrane. **(c)** Internal electrolyte leakage data for the membranes as a function of time.

As most commercial REs use a liquid-type solution as an internal electrolyte, which can leak under various pressures and salinity conditions, periodic replacement and maintenance are necessary to retain the electrode performance (Kim et al., 2017). Compared to the liquid-filled electrodes, gel-type internal electrolytes have numerous advantages. It is possible to take measurements with various pressure changes using gel-type electrolytes, and these are heat resistant up to 121°C (Guth et al., 2009; Inzelt et al., 2013). Various water-soluble polymers such as polyvinyl alcohol, methylcellulose, and HEC can be used as gel-type internal electrolytes owing to their viscous characteristics. These play a key role in preventing electrolyte leakage from the internal to external electrodes. Among these polymers, HEC is a nonionic cellulose, the aqueous solutions of which are stable at pH 2–12 with the suitable solution viscosities and gelling states at normal temperatures. HEC also exhibits a high salt tolerance and long-term stability over a wide pH range (3.5–11). For this reason, HEC can be applied to gel-type internal electrolytes for application in coastal structures. Figure 2A shows the relationship between the HEC content, viscosity, and ion conductivity. The results reveal that the viscosity of each electrolyte increases upon the addition of HEC, and a maximum value is obtained at 8 wt.% HEC. The viscosity of the electrolyte with >10 wt.% HEC cannot be measured with the viscometer owing to its limited detection range (1.5–4,800 cP). In contrast, the ion conductivity of the electrolyte decreases with an increase in the HEC content because of the limitation of ion mobilities. The time dependency of ion conductivity for the as-synthesized gel-type electrolytes is shown in Figure 2B. The ion conductivities of all internal electrolytes with different HEC contents stabilize after initial measurements (1–3 days), except with high HEC contents (12 and 15 wt.%) owing to ion transfer limitations. The gel-type electrolyte with 5 wt.% HEC shows higher ion conductivity and appropriate viscosity compared with those of other samples. Based on these results, the electrolyte with 5 wt.% HEC was selected as the optimal internal electrolyte for fabricating the Ag/AgCl ISE. Figure 2C shows the images of the gel-type internal electrolyte, acquired 3 s after electrolyte synthesis. The data show that gelling progresses with an increase in the HEC content. In particular, ideal gelling characteristics are observed with 12 and 15 wt.% HEC. The membrane is a critical part of the electrode. It not only ensures the transmission of electrolyte but also decreases it and determines the lifetime of the electrode. The FE-SEM images of two types of membranes employed are shown in Figures 3a,b. The particle size of the DCM is smaller than that of the Vycor glass membrane (VGM); the average particle sizes are 1.5 and 5.4 μm, respectively. The small particle size of DCM with a dense structure can minimize the leakage of internal electrolyte from the electrode, which can lead to the stability of the electrode in RC structures. Additionally, the morphology of VGM shows a randomly stacked plate-type structure with large pore sizes, which leads to the leakage of the internal electrolyte. Figure 3c shows the time-dependent leakage of the internal electrolyte from the PVC housing with four types of membranes. After 1 month, electrolyte leakage is observed in all membranes, which decreases with an increase in the ceramic content. However, the two types of DCMs show lower leakages compared to those of VGMs. These results are in good agreement with the corresponding FE-SEM data. In this study, GP and immersion were used to synthesize AgCl-coated Ag wire to remove undesired impurities from the Ag wire surface and increase the active sites using small AgCl particles for enhancing the interaction between Cl^−^ and the as-synthesized Ag/AgCl wire by introducing fine roughness. For this process, the WE terminal of the potentiostat was connected to the Pt-mesh and the CE terminal was connected to the Ag wire. In this case, the immersion process after GP is important for the formation of the AgCl layer on the Ag wire surface. The suggested formation mechanism of the AgCl layer on the Ag wire during GP and immersion is described by reactions (1) and (2).

(1) $$\begin{array}{c} \text{C}{\text{l}}_{2\left(\text{g}\right)}+{\text{H}}_{2}{\text{O}}_{\left(\text{aq}\right)}\to \text{HOC}{\text{l}}_{\left(\text{aq}\right)}+\text{HC}{\text{l}}_{\left(\text{aq}\right)} \end{array}$$

(2) $$\begin{array}{cc} 2\text{A}{\text{g}}_{\left(\text{s}\right)}+\text{HOC}{\text{l}}_{\left(\text{aq}\right)}\to \text{AgC}{\text{l}}_{\left(\text{s}\right)}+\text{AgO}{\text{H}}_{\left(\text{aq}\right)}\text{ΔG}= & \\ -153.69\text{KJ}/\text{mol} \end{array}$$

**Figure 4:**
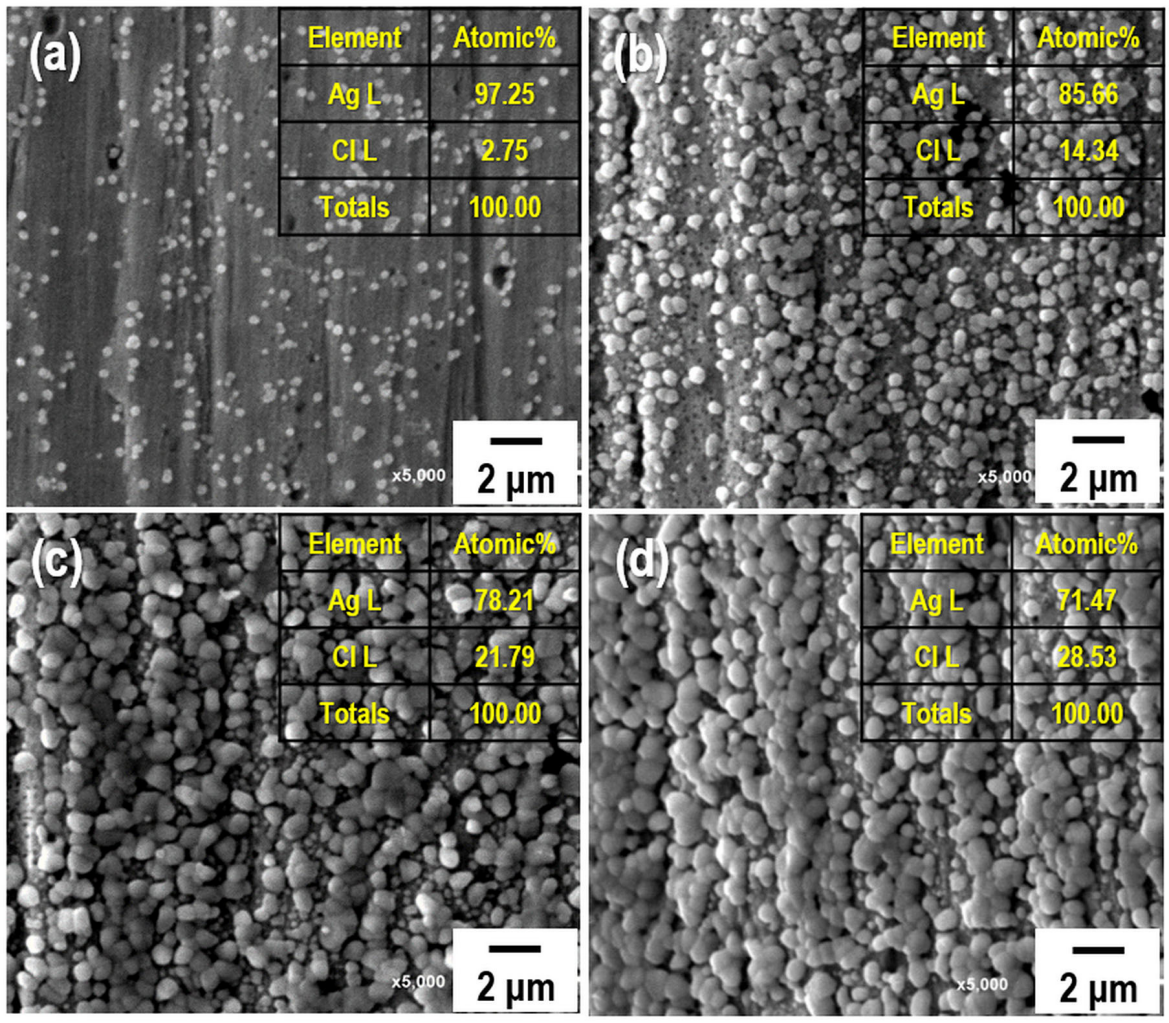
Field-emission scanning electron microscopy (FE-SEM) images and elemental compositions of the AgCl-coated Ag wire with different immersion times: **(a)** 1 s, **(b)** 60 s, **(c)** 90 s, and **(d)** 120 s. The current density and treatment time for the galvanostatic polarization of all samples are fixed at 30 mA/cm^2^ and 2 h, respectively.

**Figure 5:**
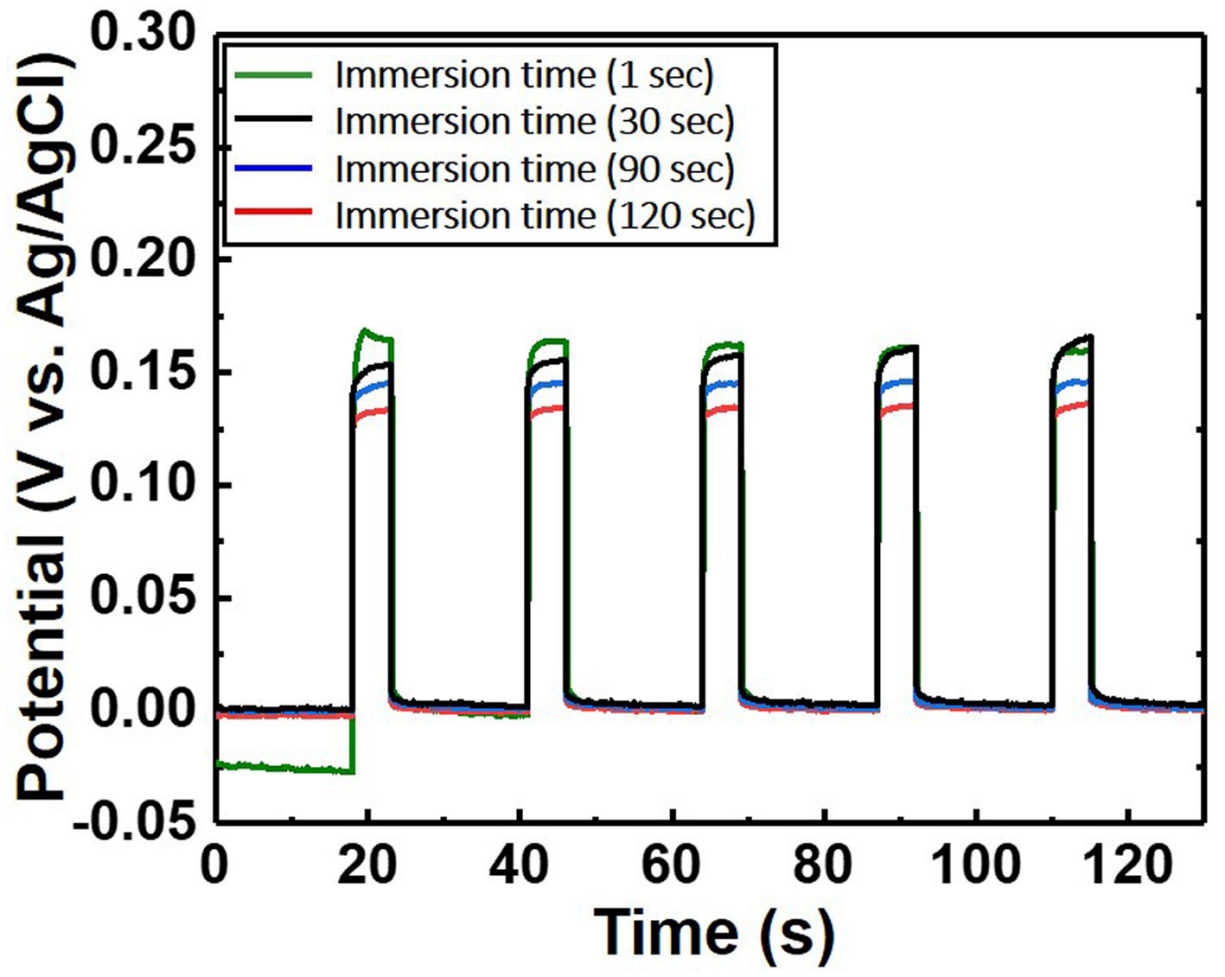
Potential difference of the chloride ion sensor as a function of immersion time. The electrolyte comprises 300 mM KCl and 0.5 M KNO_3_. The length of the Ag/AgCl wire and the distance between the Ag/AgCl wire and diatomite ceramic membrane are fixed at 3 and 1 cm, respectively.

**Figure 6:**
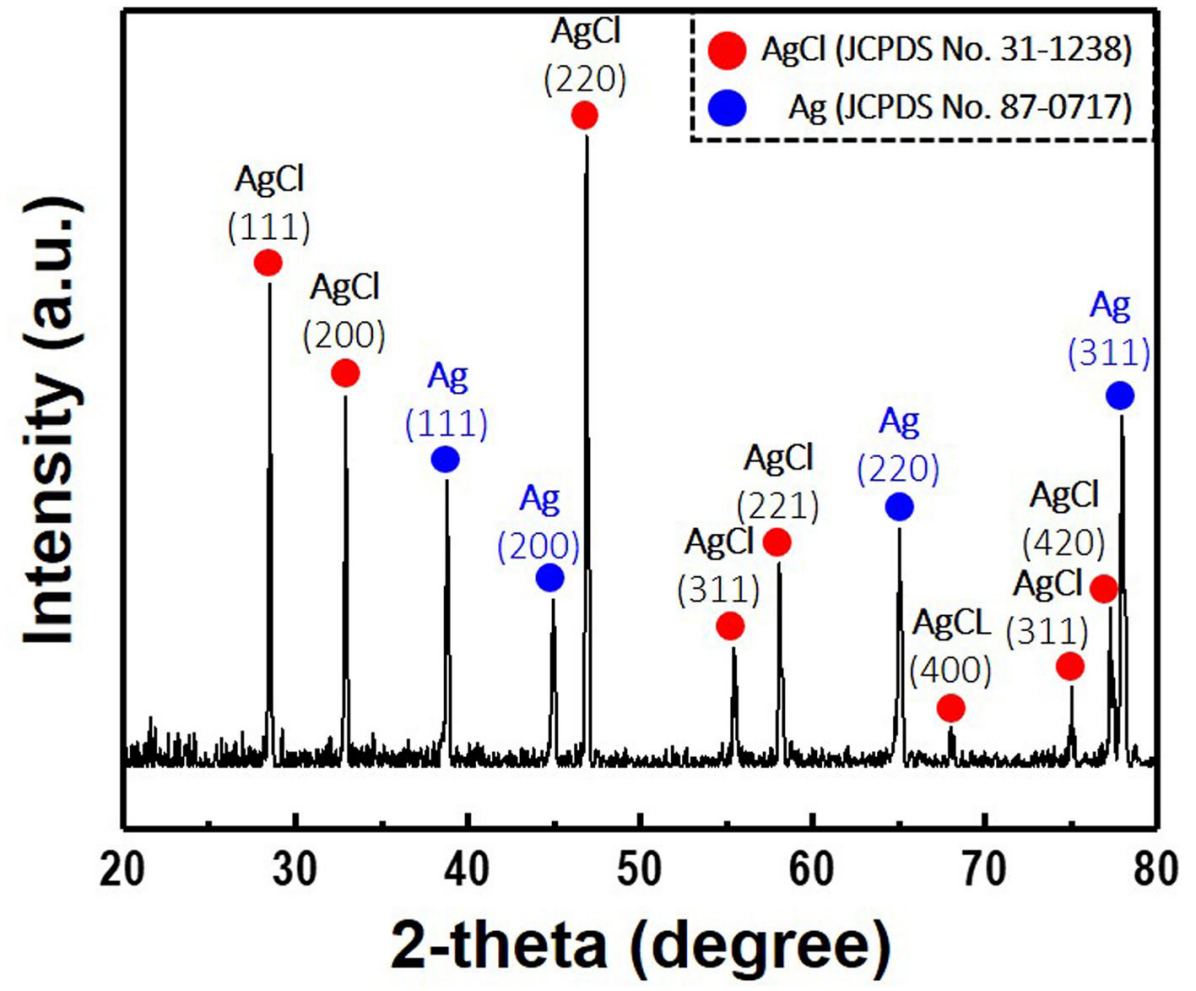
X-ray diffraction patterns of the Ag/AgCl wire synthesized via galvanostatic polarization and immersion (120 s) in 1 M hydrochloric acid. Current density and treatment time for the galvanostatic polarization are fixed at 30 mA/cm^2^ and 2 h, respectively.

**Figure 7:**
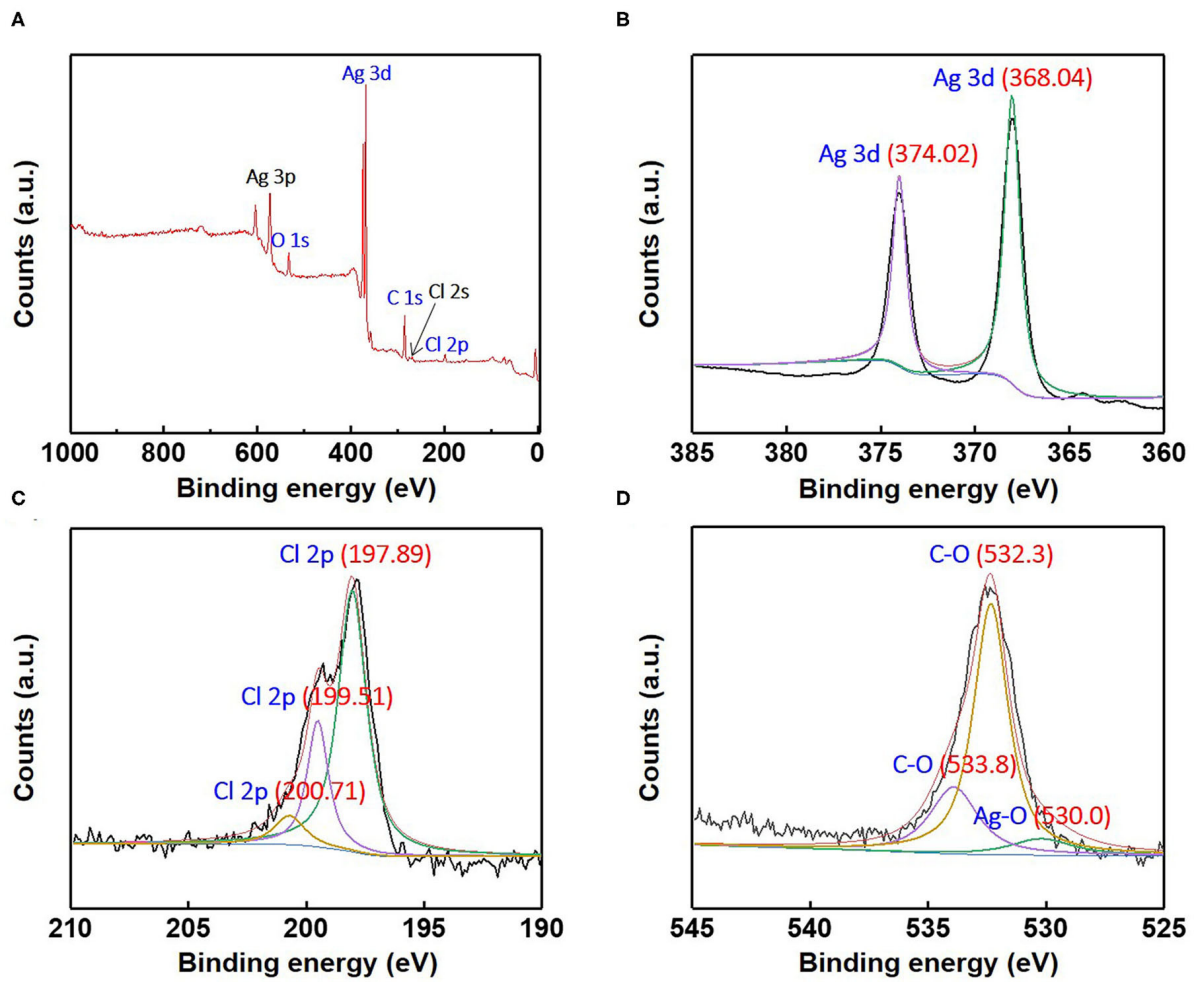
**(A)** X-ray photoelectron survey spectra and high-resolution X-ray photoelectron; **(B)** Ag3d, **(C)** Cl2p, and **(D)** O1s spectra for the as-synthesized Ag/AgCl wire.

**Figure 8:**
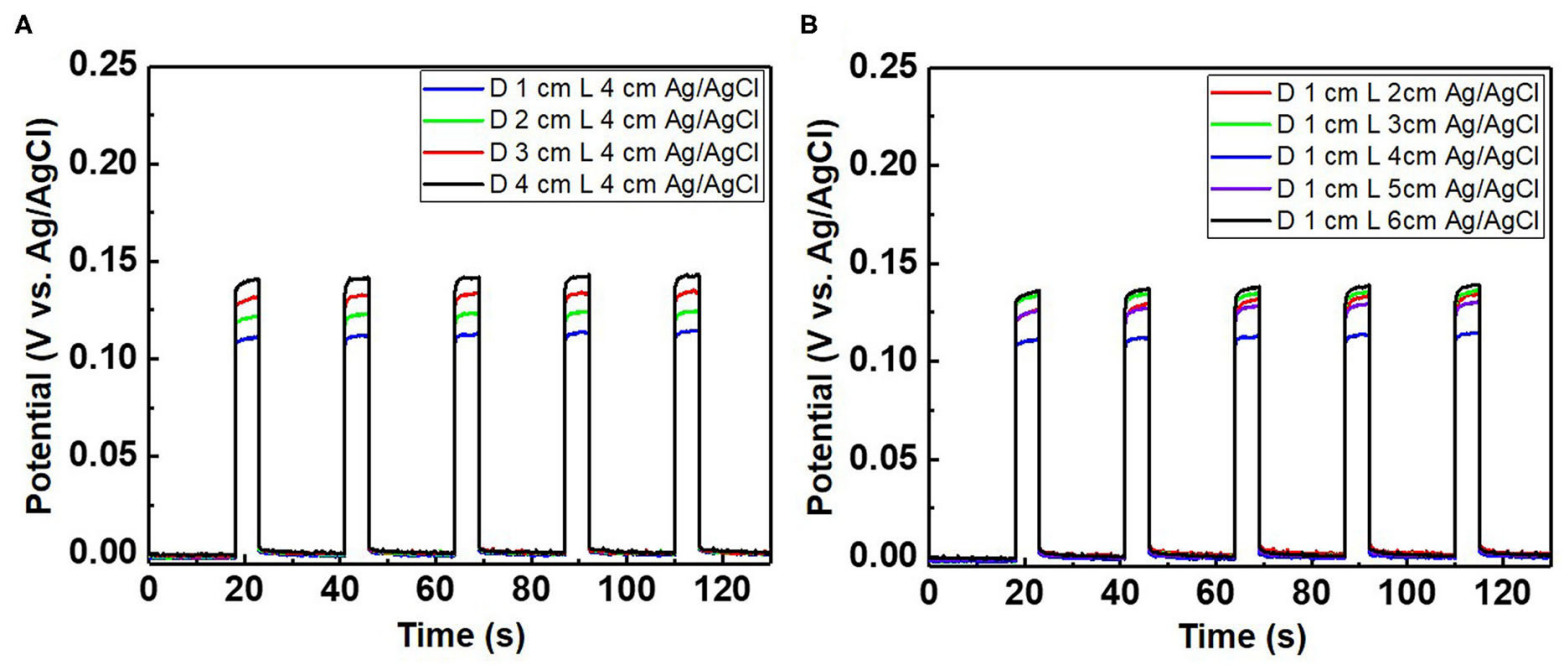
Potential difference of the chloride ion sensor as a function of **(A)** the distance (D) between the Ag/AgCl wire and diatomite ceramic membrane with a fixed wire length (L) of 4 cm and **(B)** various wire lengths with a fixed distance between the Ag/AgCl wire and diatomite membrane. The applied current pulse for chloride sensing is fixed at 1 mA, and the electrolyte comprises 300 mM KCl and 0.5 M KNO_3_.

**Figure 9:**
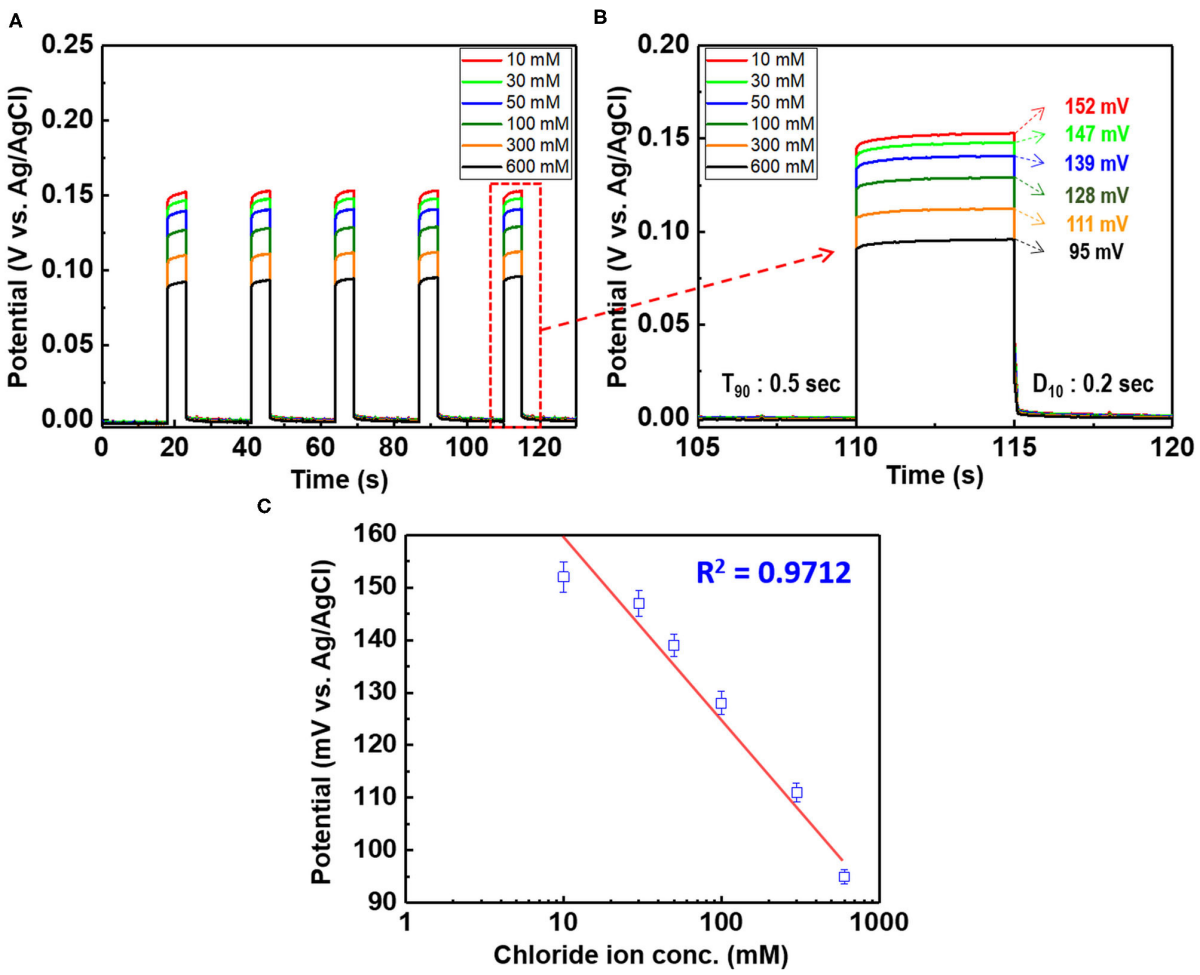
**(A)** Potential difference of the chloride ion sensor (D: 1 cm, L: 4 cm Ag/AgCl) as a function of the chloride ion content of 10–600 mM KCl and **(B)** magnified image of the fifth bar (enclosed in red rectangle) in **(A)**. **(C)** Linear fitting of the chloride ion sensor potential with the experimental data. The applied current pulse is fixed at 1 mA.

**Figure 10:**
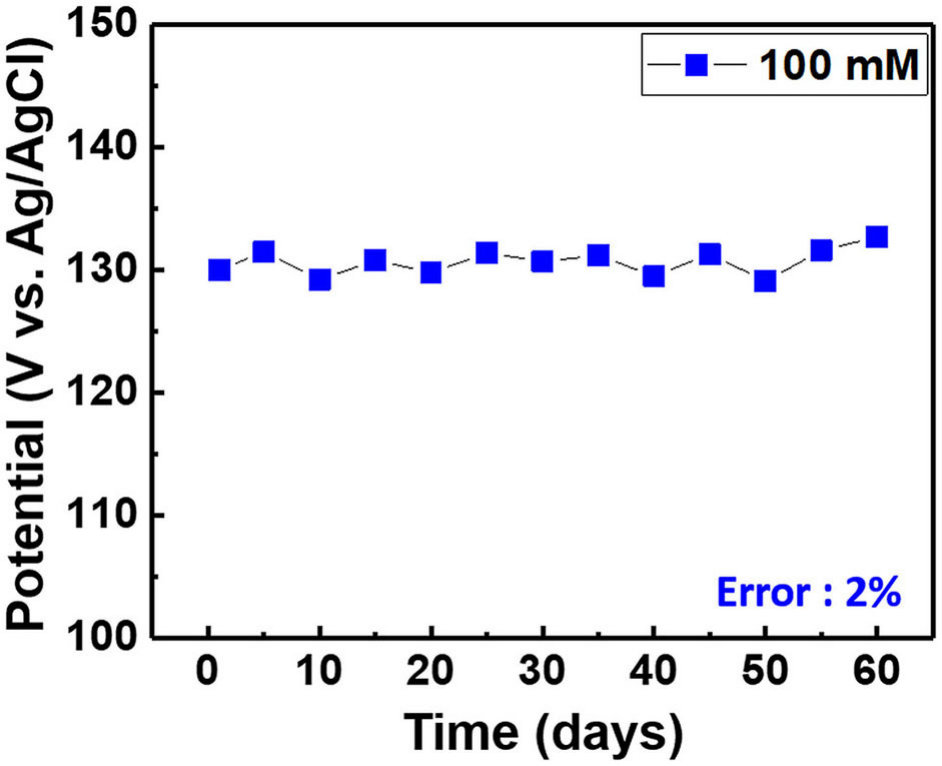
Long-term stability of the chloride ion sensor in the electrolyte comprising 100 mM KCl and 0.5 M KNO_3_.

**Figure 11:**
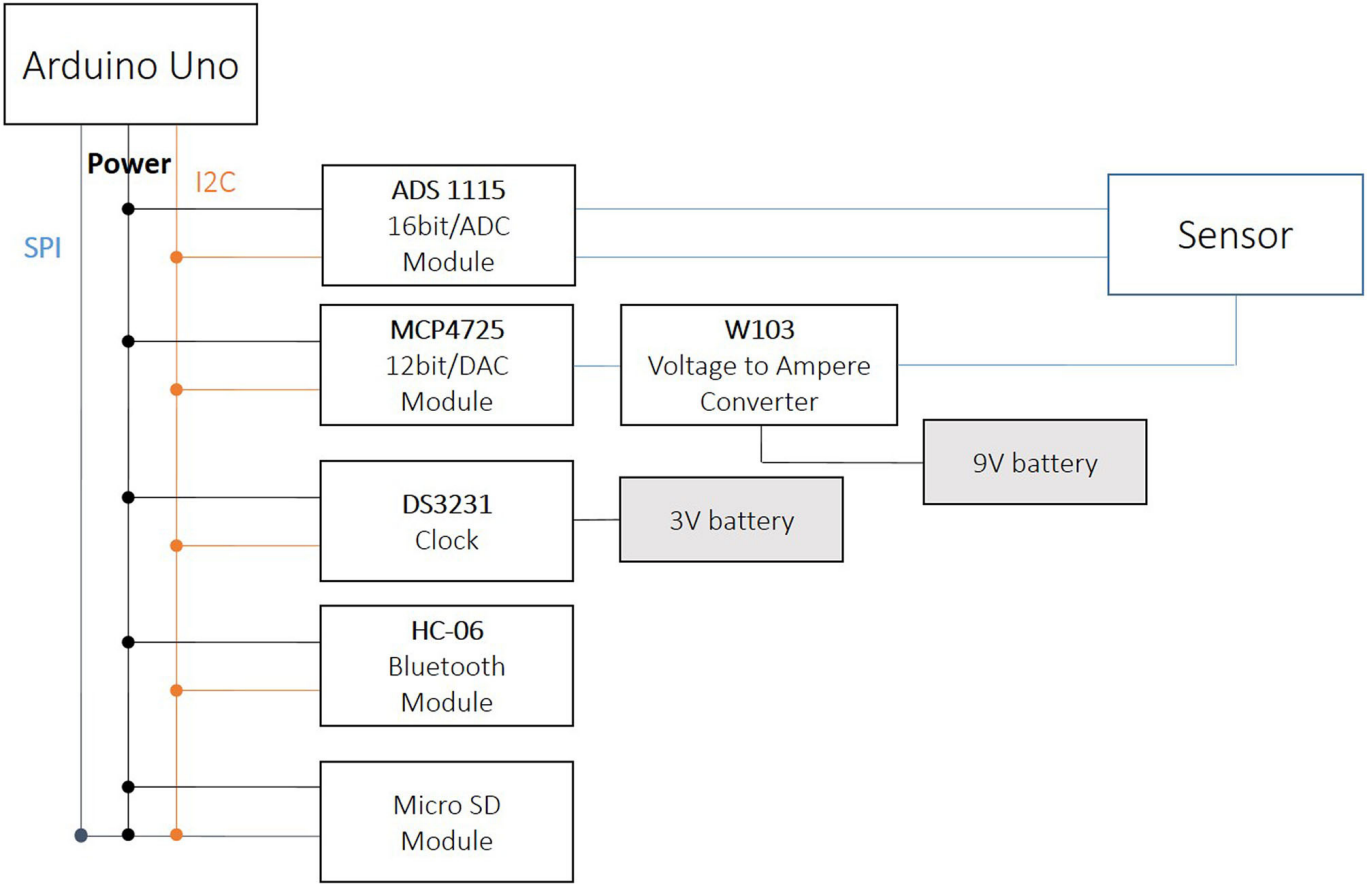
Schematic of the wireless chloride ion sensing system based on Arduino Uno.

**Figure 12:**
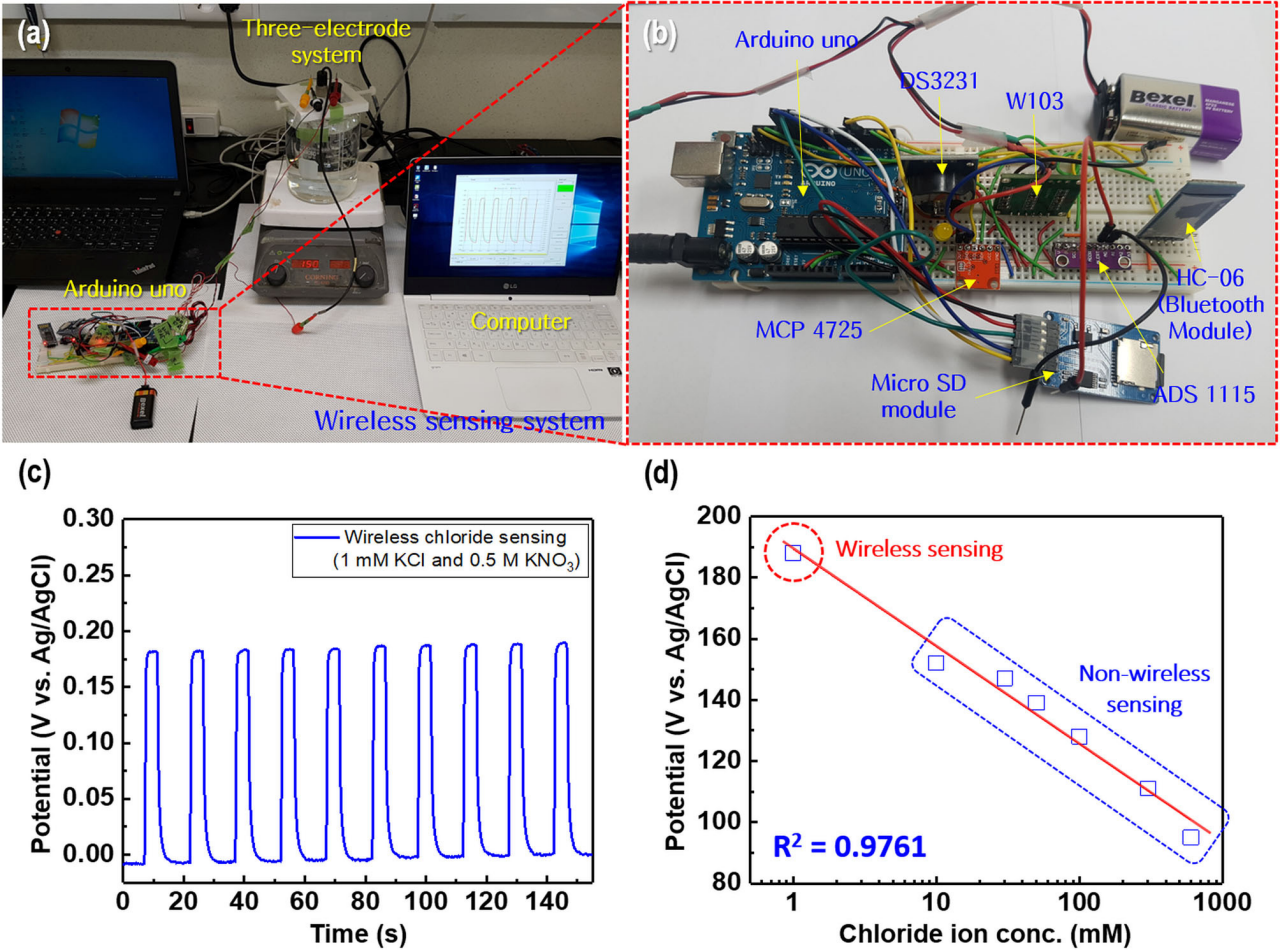
**(a)** Wireless chloride ion sensing system based on Arduino Uno and **(b)** magnified image of the wireless sensor module. **(c)** Potential difference for the wireless chloride ion sensor with a regular current pulse of 1 mA in the electrolyte comprising 1 mM KCl and 0.5 M KNO_3_. **(d)** Linear fitting of the chloride ion sensor potential response to the merged experimental data of wireless and non-wireless sensing.

When a current pulse is applied, chlorine (Cl_2_) is generated on the Pt-mesh (anode) and it reacts with water, resulting in the formation of a mixture of hypochlorous acid (HOCl) and HCl. In other words, the HOCl content in the electrolyte increases with an increase in the GP time. In this reaction, Cl_2_ is oxidized as well as reduced (Cl${}_{2}^{0}$ + H_2_O → H^+^Cl^−^ + H^+^O^2−^Cl^+^); such reactions are called auto oxidation–reduction (redox) reactions (disproportionation reaction) (Gu and Bennion, 1977; Guo et al., 2014) and involve the reduction and oxidation reactions of the same substance (reactant). In HOCl, the oxidation state of chlorine is +1, showing that it has a high affinity for electrons. Therefore, HOCl oxidizes Ag to AgCl. After GP, the immersion process is immediately carried out in the same electrolyte without applying a current pulse. When the applied current pulse is removed, the as-generated HOCl reacts with the Ag wire (cathode), resulting in the generation of AgCl and AgOH on the Ag wire surface. Figure 4 shows the FE-SEM images and elemental compositions of Ag coated with AgCl with different immersion times. The applied current density and GP time for all samples were fixed at 30 mA/cm^2^ and 2 h, respectively. As a result, the number and sizes of the as-synthesized AgCl particles, as well as the chloride content on the surface of the Ag wire, increase with an increase in immersion time. The AgCl particles appear to be relatively uniform with round shapes of <1-μm sizes and a distinct boundary between these particles in all conditions. The boundaries between AgCl particles and pore channels through the layer, known as micro-channels, are the main pathways for the transport of ions within the AgCl layer. As the AgCl layer thickness increases, the ion transport becomes limited due to micro-channel confinement (Pemberton and Girand, 1987; Neary and Parkin, 2015; Pargar et al., 2018). For this reason, it is important to optimize the applied current density and treatment time during anodization. Additionally, the immersion time is a more important parameter in the synthesis of AgCl layer on Ag wire in comparison to the current density and GP time. Cl^−^ sensing with the as-fabricated Ag/AgCl ISE was performed to confirm the effect of immersion time and determine the optimal conditions for Cl^−^ sensing by the sensor. The length of the Ag/AgCl wire and distance to the diatomite membrane were fixed at 3 and 1 cm, respectively. The Cl^−^ response was measured using a three-electrode system with the as-fabricated Ag/AgCl ISE, Pt-mesh, and a commercially available Ag/AgCl RE at ambient atmosphere (24°C) and humidity of 50%. The time periods for each applied current pulse and no-pulse were fixed at 5 and 20 s, respectively. The data for Cl^−^ sensing by the sensor in an electrolyte containing 300 mM KCl and 0.5 mM KNO_3_ as a function of immersion time using an applied current pulse of 1 mA are shown in Figure 5. For an immersion time of 1 s, the initial potential difference before applying the current pulse is ca. −0.018 V, which is ideally not zero. This result is attributed to an insufficient AgCl layer on the surface of the Ag wire, which results in different half-cell potentials with respect to the Ag/AgCl RE. Furthermore, when the current pulse is applied, the initial potential difference for an immersion time of 1 s is higher and shows a non-stable sensing signal as compared to those observed with other conditions. This is due to the formation of AgCl salt on the surface of the Ag wire during the current pulse application, resulting in the depletion of Cl^−^ near the WE and generation of a large potential difference. An immersion time of 120 s shows a lower potential difference than those obtained with 1, 60, and 90 s. As the immersion time increases, the AgCl content on Ag wire increases, while the exposed Ag surface decreases. This results in a lower depletion of Cl^−^ near the WE during Cl^−^ sensing, resulting in a low potential difference between the Ag/AgCl ISE and Ag/AgCl RE. In other words, the concentration gradient of Cl^−^ at an immersion time of 120 s is small owing to the large amount of AgCl on the Ag wire. This is in good agreement with the FE-SEM data shown in Figure 4. Therefore, an immersion time of 120 s was selected in this study as the optimal condition for synthesizing the Ag/AgCl ISE. Figure 6 shows the XRD data of the Ag/AgCl wire synthesized employing an immersion time of 120 s. It clearly confirms that the wire is composed of cubic AgCl (JCPDS No. 31-1238) and metallic Ag (JCPDS No. 04-0783). The diffraction peaks at 27.86°, 32.23°, 46.24°, 54.83°, 57.48°, 67.46°, and 76.59° correspond to the (111), (200), (220), (311), (221), (400), and (420) planes, respectively, of the Ag/AgCl layer. The peaks at 38.08°, 44.29°, 64.50°, and 77.89° are attributed to the diffraction of the (111), (200), (220), and (311) planes, respectively, of metallic Ag (Liu et al., 2019). The elemental compositions and surface chemical states of the as-synthesized Ag/AgCl wire were analyzed using XPS (Figure 7). Figure 7A shows the XPS survey spectra, indicating that Ag and Cl are the main components, and trace amounts of C and O are attributed to the adventitious hydrocarbons from the XPS instrument. The peaks at 368.04 and 374.02 eV correspond to the binding energies of Ag3d _5/2_ and Ag3d _3/2_, respectively, of Ag^+^ in AgCl (Figure 7B) (Liu et al., 2019). The two peaks at 197.89 and 199.51 eV with a doublet separation of 1.7 eV in the Cl2p spectrum correspond to the binding energies of Cl2p_3/2_ and Cl2p_1/2_, respectively (Figure 7C) (Wang et al., 2012). The results obtained from the XPS data confirm the presence of Ag and AgCl in the wire, which are in complete agreement with the XRD data. High-resolution O1s spectra of the Ag/AgCl wire include three peaks centered at 530.0, 532.3, and 533.8 eV. The latter two peaks are attributed to the organic contamination absorbed on the surface of the Ag wire, whereas the first peak located at a low binding energy is associated with Ag-O (Figure 7D) (Wang et al., 2012). The elemental atomic percentages derived from the XPS survey spectra are also shown in Table 1. These data are in good agreement with the EDS data shown in Figure 4d. To optimize the length of the Ag/AgCl wire and distance between the Ag/AgCl wire and diatomite membrane, Cl^−^ sensing by the as-fabricated Ag/AgCl ISE was investigated as a function of the distance at a fixed Ag/AgCl wire length of 4 cm (Figure 8A). As shown, the potential difference decreases with an increase in the distance between the Ag/AgCl wire and DCM. This is probably due to the low reaction speed with an increase in the distance between the Ag/AgCl wire and Cl^−^ in the electrolyte upon the application of a current pulse. Next, Cl^−^ sensing was analyzed as a function of the Ag/AgCl wire length at a fixed distance of 1 cm (Figure 8B). As observed, the Ag/AgCl wire with a length of 4 cm and distance of 1 cm shows the lowest potential difference. Based on the above experimental results, an Ag/AgCl length of 4 cm and distance of 1 cm to the membrane were selected to fabricate the Cl^−^ sensor. The potential difference of the Cl^−^ sensor as a function of Cl^−^ concentration with an identical current pulse of 1 mA is shown in Figure 9A. Prior to the application of the current pulse, the potential difference is nearly zero for all Cl^−^ concentrations because of the nearly identical half-cell potential between the as-fabricated Ag/AgCl ISE (WE) and commercially available Ag/AgCl RE. Figure 9B shows the magnification of the fifth data set (enclosed in red rectangle) in Figure 9A. With an increase in the amount of Cl^−^ in the electrolyte, the potential difference between the Ag/AgCl ISE and Ag/AgCl RE decreases. This is due to ion depletion near the WE in the electrolyte with a high Cl^−^ concentration compared to the low-concentration electrolyte. The response and recovery times were calculated using the time required for 90% change in the potential difference upon the supply and removal of Cl^−^, respectively (Kwon et al., 2016). The average response and recovery times of the Cl^−^ sensor were 0.5 and 0.1 s, respectively. When no kinetic restrictions are applied, the dynamic equilibrium between metallic silver (Ag^0^) and Ag^+^ can be established in a short duration. This feature makes the noble Ag metal prone to “corrosion” in environments with aggressive ions such as Cl^−^ (Pargar et al., 2017). As shown in Figure 9C, a good linear fit curve is obtained, and the correlation coefficient (*R*^2^) of the Cl^−^ sensor is 0.9712. As *R*^2^ value approaches 1, a strong relationship between the sensing signal and Cl^−^ concentration is observed (Kim et al., 2019). The long-term stability of the Cl^−^ sensor is important for ensuring excellent and reliable sensing performance. The potential difference of the sensor was measured as a function of time over a period of 60 days at 5-day intervals using 100 mM KCl solution (Figure 10). The relative error between the initial and final signals was 2%, indicating that the potential response was quite stable during the test period. A wireless Cl^−^ monitoring system based on Arduino Uno was developed in this study for the real-time monitoring of Cl^−^ in the electrolyte comprising 1 mM KCl and 0.5 M KNO_3_. Schematic and real images of the wireless sensing system are shown in Figures 11, 12a,b, respectively. The wireless Cl^−^ sensing system is composed of individual electronic components including the micro controller unit, voltage to ampere converter (W103), clock (DS 3231), micro SD module, as-fabricated Cl^−^ sensor, circuit device for the wireless sensing measurement of the contained single channel readout, Arduino Uno, and analysis software (visual basic). Wireless Cl^−^ sensing characterization of the sensor was performed to measure the repeated potential responses in Cl^−^-containing solution. The potential difference for the Cl^−^ sensor with a regular current pulse of 1 mA is shown in Figure 12c. The 10 reversible cycles of the response curve indicate the stable and repeatable response characteristics with a small relative error (<4%) between the average initial and final signals. Linear fit curves of the Cl^−^ sensor potential response to the merged experimental data of wireless and non-wireless sensing are shown in Figure 12d, the *R*^2^ of the Cl^−^ sensor is 0.9761. It was found that the wireless sensing of Cl^−^ sensor is reliable data. A movie for the real-time wireless Cl^−^ sensing with an applied current pulse of 1 mA in 1 mM KCl and 0.5 M KNO_3_ is shown in Supplementary Figure 1.

**Table 1:** Atomic percentage of Ag/AgCl wire determined from X-ray photoelectron spectroscopy (XPS) survey spectra after galvanostatic polarization and immersion in 1 M hydrochloric acid.

	**Ag3d**	**O1s**	**Cl2p**	**C1s**
Atomic percentage (at.%)	6.85	6.8	2.88	30.28

## Conclusion

A novel Cl^−^ sensor based on Ag wire coated with an AgCl layer composed of a gel-type internal electrolyte and DCM was manufactured *via* GP and immersion in 1 M HCl solution. Both the DCM and the gel-type electrolyte played essential roles in preventing electrolyte leakage from the ion-selective electrode. The AgCl layer was dense and uniformly coated on the surface of the Ag wire; the AgCl particles were relatively uniform with round shapes and a size of <1 μm. The size and content of AgCl increased with an increase in the immersion time at a fixed current density of 30 mA/cm^2^ and GP time of 2 h. The length of the Ag/AgCl wire and its distance to the membrane were optimized to minimize the potential difference. Consequently, the sensor could detect Cl^−^ in 10–500 mM Cl^−^ concentration with good linearity; moreover, the sensor exhibits good long-term stability (about 2 months). A wireless sensing system based on Arduino was investigated to measure the Cl^−^ response in an electrolyte containing 1 mM KCl and 0.5 M KNO_3_. A small relative error of <4% was obtained between the initial and final signals. A linearity of 0.9712 was obtained from the experimental data of non-wireless Cl^−^ sensing. In conclusion, this study presented a practical method for inexpensive and scalable fabrication of wireless Ag/AgCl-based Cl^−^ sensors for applications in RC structures with good reliability.

## Data Availability Statement

The original contributions presented in the study are included in the article/Supplementary Material, further inquiries can be directed to the corresponding author/s.

## Author Contributions

SK and GP conducted the most experiment and wrote the paper. H-JA, BY, and I-HS made some parts and did analysis. K-HL made Arduino set-up. J-HL and J-YL organized the experiment and revised the manuscript. All authors contributed to the article and approved the submitted version.

## Conflict of Interest

The authors declare that the research was conducted in the absence of any commercial or financial relationships that could be construed as a potential conflict of interest.

## Supplementary Material

Video S1 The movie of wireless chloride ion sensing based on Arduino Uno.
